# Supplementary material for: Fasting Glucose Level on the Oral Glucose Tolerance Test Is Associated with the Need for Pharmacotherapy in Gestational Diabetes Mellitus
Source: Nutrients. 2023 Feb 28;15(5):1226. doi: 10.3390/nu15051226 (PMC10005728; doi:10.3390/nu15051226)
Supplement: Supplementary file 1 [file nutrients-15-01226-s001.zip › nutrients-2218722-supplementary.pdf]

**Supplementary Table S1.** Hazard ratio for medication by glucose tolerance test result categories.

| | Unadjusted | | Adjusted * | |
|---|---|---|---|---|
| | **HR** | **95% CI** | **HR** | **95% CI** |
| Fasting OGTT | | | | |
| <4.3 | 1.00 | | 1.00 | |
| 4.3-<4.6 | 1.77(1.13–2.77) | | 1.64(1.05–2.57) | |
| 4.6-<5.1 | 2.89(1.93–4.33) | | 2.62(1.74–3.95) | |
| 5.1-<5.4 | 4.39(2.84–6.76) | | 3.22(2.03–5.10) | |
| 5.4+ | 5.88(3.76–9.18) | | 3.90(2.41–6.31) | |
| One-hour OGTT | | | | |
| <8.9 | 1.00 | | 1.00 | |
| 8.9-<10.0 | 1.04(0.68–1.58) | | 1.38(0.89–2.16) | |
| 10.0-<10.8 | 1.15(0.78–1.71) | | 1.34(0.90–2.01) | |
| 10.8+ | 1.81(1.24–2.63) | | 2.15(1.46–3.17) | |
| Two-hour OGTT | | | | |
| <8.0 | 1.00 | | 1.00 | |
| 8.0-<8.7 | 0.49(0.35–0.69) | | 0.65(0.44–0.95) | |
| 8.7-<9.4 | 0.64(0.46–0.88) | | 0.81(0.57–1.16) | |
| 9.4+ | 0.80(0.58–1.10) | | 1.00(0.71–1.40) | |
| Combined | | | | |
| Fasting and 1 hr or 2 hr | 4.02(2.88–5.61) | | 2.96(2.02–4.34) | |
| Fasting only | 2.68(1.88–3.82) | | 2.07(1.37–3.14) | |
| 1 hr and 2 hr only | 1.82(1.30–2.56) | | 1.98(1.38–2.84) | |
| 1 hr only | 1.26(0.84–1.90) | | 1.30(0.84–2.02) | |
| 2 hr only | 1.00 | | 1.00 | |

* Adjusted for ethnicity (Caucasian, South Asian, South-East Asian, Other), birth year (in categories), parity (1, 2, 3+), maternal age (centred continuous), BMI (centred continuous).

**Supplementary Table S2.** Hazard ratio for medication by glucose tolerance test result categories.

| | Unadjusted | | Adjusted * | |
|---|---|---|---|---|
| | **HR** | **95% CI** | **HR** | **95% CI** |
| Fasting OGTT | | | | |
| <4.3 | 1.00 | | 1.00 | |
| 4.3-<4.6 | 1.77(1.13–2.77) | | 1.64(1.05–2.57) | |
| 4.6-<5.1 | 2.89(1.93–4.33) | | 2.62(1.74–3.95) | |
| 5.1-<5.4 | 4.39(2.84–6.76) | | 3.22(2.03–5.10) | |
| 5.4+ | 5.88(3.76–9.18) | | 3.90(2.41–6.31) | |
| One-hour OGTT | | | | |
| <8.9 | 1.00 | | 1.00 | |
| 8.9-<10.0 | 1.04(0.68–1.58) | | 1.38(0.89–2.16) | |
| 10.0-<10.8 | 1.15(0.78–1.71) | | 1.34(0.90–2.01) | |
| 10.8+ | 1.81(1.24–2.63) | | 2.15(1.46–3.17) | |
| Two-hour OGTT | | | | |
| <8.0 | 1.00 | | 1.00 | |
| 8.0-<8.7 | 0.49(0.35–0.69) | | 0.65(0.44–0.95) | |
| 8.7-<9.4 | 0.64(0.46–0.88) | | 0.81(0.57–1.16) | |
| 9.4+ | 0.80(0.58–1.10) | | 1.00(0.71–1.40) | |
| Combined | | | | |
| Fasting and 1 hr or 2 hr | 4.02(2.88–5.61) | | 2.96(2.02–4.34) | |
| Fasting only | 2.68(1.88–3.82) | | 2.07(1.37–3.14) | |

| | | |
|---|---|---|
| 1 hr and 2 hr only | 1.82(1.30–2.56) | 1.98(1.38–2.84) |
| 1 hr only | 1.26(0.84–1.90) | 1.30(0.84–2.02) |
| 2 hr only | 1.00 | 1.00 |

* Adjusted for ethnicity (Caucasian, South Asian, South-East Asian, Other), birth year (in categories), parity (1, 2, 3+), maternal age (centred continuous), BMI (centred continuous).

**Supplementary Table S3.** GDM diagnostic group and third trimester foetal ultrasound.

| Outcome | | | GA at Scan | EFW | AC |
|---|---|---|---|---|---|
| Fasting only | *n* = 77 | Mean | 31.10 | 2084.00 | 29.00 |
| | | SD | 3.10 | 682.00 | 3.40 |
| | | Missing (*n*) | 42.00 | 32.00 | 33.00 |
| Fasting and 1 hr or 2 hr | *n* = 91 | Mean | 32.00 | 2240.00 | 29.50 |
| | | SD | 3.60 | 807.00 | 4.30 |
| | | Missing (*n*) | 47.00 | 32.00 | 32.00 |
| 1 hr and 2 hr only | *n* = 113 | Mean | 32.70 | 2226.00 | 29.60 |
| | | SD | 3.30 | 652.00 | 3.90 |
| | | Missing (*n*) | 68.00 | 60.00 | 59.00 |
| 1 hr only | *n* = 78 | Mean | 31.70 | 2265.00 | 29.90 |
| | | SD | 3.10 | 732.00 | 3.70 |
| | | Missing (*n*) | 48.00 | 43.00 | 43.00 |
| 2 hr only | *n* = 295 | Mean | 32.10 | 2171.00 | 29.20 |
| | | SD | 3.40 | 730.00 | 3.80 |
| | | Missing (*n*) | 175.00 | 146.00 | 144.00 |
| | ANOVA | *p* Value | 0.02 | 0.50 | 0.50 |

**Supplementary Table S4.** Hazard ratio for outcomes by diagnostic criteria.

| Outcome | Fasting only (ref) | Fasting and 1 hr or 2 hr | | 1 hr and 2 hr only | | 1 hr only | | 2 hr only | |
|---|---|---|---|---|---|---|---|---|---|
| Preterm births (<37 weeks) | | | | | | | | | |
| Unadjusted | 1.00 | 0.86 | (0.21–3.43) | 1.92 | (0.61–6.03) | 1.49 | (0.42–5.25) | 1.53 | (0.53–4.40) |
| Adjusted Model 1 * | 1.00 | 0.68 | (0.15–3.08) | 2.06 | (0.59–7.14) | 1.59 | (0.46–5.50) | 1.77 | (0.58–5.42) |
| Adjusted Model 2 ** | 1.00 | 0.70 | (0.15–3.20) | 1.94 | (0.55–6.81) | 1.48 | (0.43–5.15) | 1.56 | (0.51–4.81) |
| Early term births (<39 weeks) | | | | | | | | | |
| Unadjusted | 1.00 | 1.74 | (1.13–2.67) | 1.16 | (0.74–1.80) | 1.41 | (0.90–2.23) | 1.08 | (0.73–1.59) |
| Adjusted Model 1 | 1.00 | 1.72 | (1.09–2.71) | 1.28 | (0.79–2.09) | 1.54 | (0.96–2.48) | 1.15 | (0.74–1.79) |
| Adjusted Model 2 | 1.00 | 1.65 | (1.06–2.56) | 1.30 | (0.81–2.08) | 1.59 | (1.00–2.52) | 1.22 | (0.79–1.87) |
| Macrosomia (>4 kg) | | | | | | | | | |
| Unadjusted | 1.00 | 0.46 | (0.15–1.39) | 0.29 | (0.09–0.92) | 0.42 | (0.13–1.38) | 0.47 | (0.21–1.06) |
| Adjusted Model 1 | 1.00 | 0.51 | (0.15–1.77) | 0.35 | (0.10–1.25) | 0.56 | (0.15–2.07) | 0.71 | (0.30–1.68) |
| Adjusted Model 2 | 1.00 | 0.53 | (0.16–1.84) | 0.34 | (0.10–1.15) | 0.55 | (0.15–2.03) | 0.63 | (0.28–1.45) |
| Large for gestational age | | | | | | | | | |
| Unadjusted | 1.00 | 0.96 | (0.43–2.18) | 0.30 | (0.10–0.86) | 0.53 | (0.20–1.42) | 0.68 | (0.34–1.35) |
| Adjusted Model 1 | 1.00 | 1.14 | (0.52–2.48) | 0.29 | (0.08–1.03) | 0.70 | (0.24–2.05) | 0.93 | (0.43–2.00) |
| Adjusted Model 2 | 1.00 | 1.03 | (0.46–2.29) | 0.31 | (0.08–1.15) | 0.69 | (0.22–2.16) | 1.09 | (0.48–2.47) |
| Small for gestational age | | | | | | | | | |
| Unadjusted | 1.00 | 0.99 | (0.36–2.73) | 1.19 | (0.47–3.01) | 1.63 | (0.63–4.21) | 1.28 | (0.57–2.87) |
| Adjusted Model 1 | 1.00 | 1.16 | (0.37–3.68) | 1.17 | (0.41–3.31) | 1.81 | (0.64–5.12) | 1.26 | (0.46–3.40) |
| Adjusted Model 2 | 1.00 | 1.09 | (0.33–3.63) | 1.08 | (0.38–3.11) | 1.62 | (0.57–4.59) | 1.13 | (0.41–3.07) |
| Birth by caesarean section | | | | | | | | | |
| Unadjusted | 1.00 | 1.28 | (0.77–2.13) | 1.26 | (0.79–2.00) | 1.28 | (0.76–2.14) | 0.95 | (0.62–1.45) |
| Adjusted Model 1 | 1.00 | 1.31 | (0.76–2.27) | 1.85 | (1.07–3.19) | 1.85 | (1.05–3.27) | 1.51 | (0.92–2.49) |
| Adjusted Model 2 | 1.00 | 1.31 | (0.76–2.24) | 1.86 | (1.08–3.23) | 1.91 | (1.08–3.40) | 1.54 | (0.94–2.54) |
| Caesarean section or instrumental birth | | | | | | | | | |
| Unadjusted | 1.00 | 1.38 | (0.89–2.12) | 1.15 | (0.77–1.72) | 1.12 | (0.77–1.72) | 0.96 | (0.67–1.38) |

| | | | | | | | | |
|---|---|---|---|---|---|---|---|---|
| Adjusted Model 1 | 1.00 | 1.32 | (0.84–2.07) | 1.45 | (0.93–2.25) | 1.37 | (0.93–2.25) | 1.28 | (0.85–1.94) |
| Adjusted Model 2 | 1.00 | 1.35 | (0.86–2.13) | 1.51 | (0.96–2.35) | 1.43 | (0.96–2.35) | 1.34 | (0.88–2.03) |
| Gestational hypertension at birth | | | | | | | | | |
| Unadjusted | 1.00 | 1.52 | (0.45–5.14) | 0.87 | (0.23–3.24) | 1.76 | (0.23–3.24) | 0.65 | (0.21–2.06) |
| Adjusted Model 1 | 1.00 | 1.64 | (0.49–5.50) | 0.91 | (0.22–3.75) | 2.05 | (0.22–3.75) | 0.64 | (0.18–2.26) |
| Adjusted Model 2 | 1.00 | 1.45 | (0.43–4.87) | 0.91 | (0.22–3.66) | 2.13 | (0.22–3.66) | 0.64 | (0.19–2.22) |
| Transfer to neonatal ICU | | | | | | | | | |
| Unadjusted | 1.00 | 1.56 | (0.52–4.65) | 2.11 | (0.77–5.83) | 1.39 | (0.77–5.83) | 1.82 | (0.71–4.67) |
| Adjusted Model 1 | 1.00 | 1.59 | (0.50–5.06) | 2.62 | (0.89–7.76) | 1.75 | (0.89–7.76) | 2.41 | (0.88–6.60) |
| Adjusted Model 2 | 1.00 | 1.64 | (0.52–5.19) | 2.65 | (0.90–7.80) | 1.75 | (0.90–7.80) | 2.37 | (0.87–6.50) |
| Neonatal hypoglycaemia | | | | | | | | | |
| Unadjusted | 1.00 | 1.07 | (0.45–2.55) | 0.85 | (0.36–2.02) | 0.22 | (0.36–2.02) | 0.64 | (0.30–1.38) |
| Adjusted Model 1 | 1.00 | 1.64 | (0.62–4.31) | 1.33 | (0.53–3.33) | 0.24 | (0.53–3.33) | 1.36 | (0.57–3.25) |
| Adjusted Model 2 | 1.00 | 1.58 | (0.62–4.06) | 1.35 | (0.55–3.30) | 0.26 | (0.55–3.30) | 1.48 | (0.63–3.47) |
| Newborn respiratory distress | | | | | | | | | |
| Unadjusted | 1.00 | 0.74 | (0.27–2.05) | 0.92 | (0.37–2.28) | 0.48 | (0.37–2.28) | 0.64 | (0.28–1.45) |
| Adjusted Model 1 | 1.00 | 0.80 | (0.30–2.13) | 1.17 | (0.45–3.02) | 0.59 | (0.45–3.02) | 0.91 | (0.36–2.33) |
| Adjusted Model 2 | 1.00 | 0.81 | (0.30–2.17) | 1.18 | (0.46–3.08) | 0.59 | (0.46–3.08) | 0.93 | (0.36–2.37) |

* Adjusted for ethnicity (Caucasian, South Asian, South-East Asian, Other), birth year (in categories), parity (1, 2, 3+), maternal age (centred continuous), BMI (centred continuous). ** Adjusted as per model 1 and additionally including treatment type (diet, insulin, metformin, insulin and metformin).
